# Mortality and life expectancy trends in Spain by pension income level for male pensioners in the general regime retiring at the statutory age, 2005**–**2018

**DOI:** 10.1186/s12939-022-01697-2

**Published:** 2022-07-14

**Authors:** Juan M. Pérez-Salamero González, Marta Regúlez-Castillo, Manuel Ventura-Marco, Carlos Vidal-Meliá

**Affiliations:** 1 Department of Financial Economics and Actuarial Science, University of Valencia, Avenida de los Naranjos s.n, 46022 Valencia, Spain; 2 Department of Applied Economics III, University of the Basque Country (UPV/EHU), Avda. Lehendakari Aguirre 84, 48015 Bilbao, Spain; 3 Instituto Complutense de Análisis Económico, Complutense (ICAE), Complutense University of Madrid, Madrid, Spain

**Keywords:** Inequalities, Life expectancy, Mortality, Socioeconomic

## Abstract

**Background:**

Research has generally found a significant inverse relationship in mortality risk across socioeconomic (SE) groups. This paper focuses on Spain, a country for which there continues to be very little evidence available concerning retirement pensioners. We draw on the Continuous Sample of Working Lives (CSWL) to investigate disparities in SE mortality among retired men aged 65 and above over the longest possible period covered by this data source: 2005–2018. We use the initial pension income (PI) level as our single indicator of the SE status of the retired population.

**Methods:**

The mortality gradient by income is quantified in two ways: via an indicator referred to as “relative mortality”, and by estimating changes in total life expectancy (LE) by PI level at ages 65 and 75 over time. We show that, should the information provided by the relative mortality ratio not be completely clear, a second indicator needs to be introduced to give a broad picture of the true extent of inequality in mortality.

**Results:**

The first indicator reveals that, for the period covered and for all age groups, the differences in death rates across PI levels widens over time. At older age groups, these differences across PI levels diminish. The second indicator shows that disparities in LE at ages 65 and 75 between pensioners in the lowest and highest income groups are relatively small, although slightly higher than previously reported for Spain. This gap in LE widens over time, from 1.49 to 2.54 years and from 0.71 to 1.40 years respectively for pensioners aged 65 and 75. These differences are statistically significant.

**Conclusions:**

Along with other behavioral and structural aspects, a combination of factors such as the design of the pension system, the universality and quality of the health system, and high levels of family support could explain why LE inequalities for retired Spanish men are relatively small. To establish the reasons for this increased inequality in LE, more research needs to be carried out. An analysis of all Spanish social security records instead of just a sample would provide us with more information.

## Background

This paper deals with the association between socioeconomic (SE) status and mortality in old age. Any examination of SE status in mortality requires us to be clear about what the term actually means and what the most used indicators are. The term is used by epidemiologists, sociologists, economists and actuaries, to name just a few, to describe the class standing of an individual or group. At an operational level it is often translated into education, income, wealth or occupation, for example. At different ages, these potential operational definitions of SE status may differ in their meanings and relationships to health or mortality [1]. The most used SE indicators when examining mortality in old age are education, wealth and income [2, 3]. Education level often shows a weaker association with health in old age than other indices –such as wealth, income, tenure and deprivation [3] – and has several additional limitations [2]. Wealth provides individuals with the resources to manage emergencies, absorb economic shocks and obtain better health care than those with less wealth. It is also a cumulative measure of lifetime income in cases where a direct measure is unavailable. The main limitations to using wealth in mortality studies of old age are that researchers are concerned about the potential for a reverse correlation with poor health, it is very difficult to obtain accurate data on wealth and wealth varies widely over the life cycle.

Income is often considered to be a straightforward indicator of material resources and, indeed, it is robustly and positively associated with longevity [2]. The main advantage of using it as a measure of SE status is its greater range of variation compared with the clustering of educational attainment at the completion of high school and college. Sullivan and Von Wachter [4] argue that average earnings over a long period provide a better SE indicator than current earnings, since the latter are subject to short-term variation. In the literature, measures involving one’s financial situation are usually most strongly associated to health and mortality in old age rather than to education level and social class, for instance [3].

The link between SE status and mortality could have implications for a wide range of social security program rules [5, 6]. Many OECD countries have addressed the issue of increased longevity, usually by increasing the retirement age. However, this type of reform may lead to substantial transfers from those with shorter lifespans to those who will live longer than the average, since they do not necessarily take into account SE differences in mortality [7].

The implications of heterogeneity in longevity between SE groups as regards pension reform and scheme design are substantial because taxes/subsidies counteract the envisaged effects of (i) a closer contribution-benefit link, (ii) a later formal retirement age to address population aging, and (iii) more individual funding and private annuities to compensate for reduced public generosity [8, 9]. Differences in LE between high and low SE groups are often large and in recent years have widened further in many European countries. Such longevity gaps affect the actuarial fairness and progressivity of public pension systems [10–13].

Auerbach et al. [14] explore how growing inequality in LE affects lifetime benefits from Social Security, Medicare and other US programs and how this interacts with possible program reforms. They conclude that policymakers would do well to consider the welfare implications not only of improved longevity, but also of the increasing gap in LE by SE status. Also for the US, Reznik et al. [15, 16] examine the distributional effects of hypothetical changes in the Social Security retirement program on benefit adequacy and the economic status of future beneficiaries in the context of ongoing changes in LE and differential mortality. They assess the likely impact of these options on the benefit levels and economic status of different types of future beneficiary, such as those with low lifetime earnings. Baurin [17] analyses the relevance of using SE characteristics to differentiate between retirement ages. Using US mortality rates, he simulates the lifespan distribution both across and within SE categories and analyses their ability to predict the lifespan of individuals. His main conclusion is that SE status has relatively limited predictive power due to the huge lifespan heterogeneity “within” each SE category.

In short, the literature on the subject is growing and more researchers appear to be taking an interest in mortality and LE inequalities related to SE status and its impact on social security programs. Studies detailing income inequality in old-age LE are few and far between, yet there is evidence that high-income countries experience substantial and potentially increasing inequality in late-life longevity [18].

A substantial decline in mortality in lower SE groups has been reported in most of the European countries included in its scope [19, 20]. However, relative inequalities in mortality have increased almost universally, because percentage declines are usually smaller in lower SE groups.

The literature reviewed in this analysis (see the literature review section for details), which includes the UK [21, 22], the USA [23–25], Italy [26–28], Canada [29–31], Germany [32–34], the Netherlands [35, 36] and Sweden [18], generally indicates that inequalities in old-age mortality measured by SE status have widened in recent years.

This paper focuses on Spain, a country for which there continues to be very little evidence available concerning the link between longevity and income in older age groups [37–41] (see the literature review section for details). None of the cited papers for Spain look at the specific case of retirement pensioners; most focus on adults aged 50 and over from the general population due to the lack of information on retired individuals. To the best of our knowledge, only one paper [42] so far has examined differences in life expectancy (LE) between self-employed (SEP) and paid employee (PEP) workers when they become retirement pensioners, looking at levels of pension income using administrative data from Spanish social security records.

According to the above-cited literature [5–36], it is difficult to hide the real importance of this subject, i.e. the link between mortality and LE for retirement pensioners by pension income level. In the case of Spain, this is especially relevant given that an informal proposal has been made by the government to use LE by SE group to determine the initial amount of the retirement pension [43]. The Minister of Inclusion, Social Security and Migration provided data on mortality by income, sex, and age for the case of the USA [44], claiming that, judging by what was already known for Spain, there would be little difference between Spanish and US data. Our paper provides new evidence that inequality in mortality for retirement pensioners by pension income is actually much lower in Spain than the USA.

Given the features of the dataset used in this paper and the aims of the study, it is more important to examine mortality differences in relation to income (average earnings) rather than education level or wealth, because entitlement to contributory Social Security programs is based on income. We use pension income as the only SE indicator, given that the amount of the retirement benefit is an indicator of the pensioners’ earnings during their years of active life (or at least over the 15–25 years immediately preceding retirement).

We present results for mortality trends among male pensioners aged 65 and over since the mid-2000s.

We exclude those retirement pensioners belonging to the special system for the self-employed (SEP) because the pension rules make their benefits a poor proxy for lifetime income. Given that poor health is an important reason for early retirement, pensioners who access benefits before the statutory retirement age are also excluded, as are disability pensioners for the reasons explained in the methods section.

The mortality gradient by income is quantified in two ways. The first is via an indicator called relative mortality, which represents the ratio of the death rate of a subgroup to the death rate of the group as a whole. The second is by estimating changes in total LE by PI level at ages 65 (LE_65_) and 75 (LE_75_) over time. Both indicators are used worldwide to measure health inequalities [45]. As a supplementary approach a comparison with the mortality rate for the Spanish population is also provided. Our combined approach enables us to accurately answer four basic research questions: Are there differences in mortality between PI groups? If so, are they statistically significant? Are there differing trends in LE between PI groups that lead to a widening or narrowing of inequalities over time? Is the (informal) government proposal to use LE by SE group to determine the initial amount of retirement pension a good idea?

### Literature review

In the last two decades there has been a major increase in the availability of data linking mortality risk and measures of SE status for the elderly. The result has been a virtual flood of new empirical research showing not only that there are great inequities in the risk of death between those at the top and those at the bottom of the SE distribution, but also that the gap between them has been growing [2].

In the UK, death rates for people aged 60–89 improved for all groups between 2001 and 2015. However, the greatest improvement was among those in higher SE groups. The most advantaged fifth of older men saw a reduction in death rates of 32%, compared with 20% for the least advantaged fifth. Women in this age group experienced a reduction in death rates of 29% for the most advantaged fifth and 11% for the least advantaged fifth [21, 22]. Reports on the over-65 s in Germany [32–34] and in the Netherlands [35, 36] also show that inequalities in mortality as measured by SE status have widened in recent years.

Providing details of SE disparities in LE gains among retired German men, Wenau et al. [34] show that over the two decades studied (1997–2016), male mortality declined in all income groups in both German regions (west and east). Because mortality improved more rapidly among higher status groups, the social gradient in mortality widened. Since 1997, the distribution of pension entitlements for retired East German men has shifted substantially downwards. As a result, the impact of the most disadvantaged group on total mortality has increased and partly attenuated the overall improvement. Tetzlaff et al. [33] also report that, especially among the most elderly men, the gap between low- and high-income groups has widened over time. Among women, however, a slight reduction in inequalities was observed, driven by increases in LE in low-income groups.

Kalwij et al. [35] estimate a mortality risk model that explicitly controls for unobserved individual-specific heterogeneity (random effects) using administrative data taken from the 1996–2007 Income Panel Study of the Netherlands supplemented with data from the Causes of Death registry. They find for men and women that remaining LE at age 65 for low-income individuals as approximately 2.5 years less than that for high income individuals. For the period 1996–2016 in the Netherlands, Bär et al. [36] find that while inequalities in mortality have decreased at ages up to 65, they have increased for the oldest age groups.

For the USA, Waldron [24] finds a difference in both the level and the rate of change in improvements in mortality over time by SE status for male Social Security–covered workers. Bosley et al. [23] analyze the relationship between average indexed monthly earnings (AIME) and mortality rates for Social Security retired-worker beneficiaries. AIME are used to calculate the primary insurance amount (PIA) used to determine an individual’s Social Security benefits in the USA. The authors observe lower death rates for retired-worker beneficiaries with higher-than-average AIME levels and higher death rates for retired-worker beneficiaries with lower-than-average AIME levels. At older ages, the differences in death rates across AIME levels diminish. The trends from 1995 to 2015 show the difference in death rates across AIME levels remaining fairly steady. Goldman and Orszag [25] estimate LE at age 65 across AIME quintiles. The gap in LE at age 65 between the highest and lowest AIME quartiles increases by 81%, from 3.1 years for males born in 1928 to 5.6 years for males born in 1990. For females the gap almost doubles, from 1.7 years to 3.3 years over the same time period.

In Italy it has been found that the association between lifetime income and old age mortality risk is negative but weak across most of the income distribution. After controlling for regional differences, Belloni et al. [26] report that the income-old age mortality gradient is generally stable over time. However, the research by Lallo and Raitano [27] highlights extreme differences in mortality risks by SE status (occupational group, education and subjective household economic condition), confirms the existence of large health inequalities and strongly questions the fairness of the Italian public pension system. A more recent study on Italy by Ardito et al. [28] reveals an increasing gap in LE at 65 between income quartiles and occupational classes. By following three cohorts of workers employed in Italy during 1990–1994, 1995–1999 and 2000–2004 for 20 years, their analysis shows that the gap in life expectancy at 65 between the highest and the lowest income quartile widened by 0.7 years for men and 0.4 years for women. In the most recent cohort, remaining life expectancy at age 65 for men in the lowest-income quartile was approximately 1.8 years less than that for men with the highest incomes, while the corresponding gap for women was 0.33 years.

A comparison of annual mortality improvement rates over 15 years (1998 to 2013) for retirement beneficiaries by level of pension that was compiled by OSFIC [29] for Canada shows that, for both males and females in the 65 to 94 age group, the rates for those with pensions of less than 37.5% of the maximum (3.0% for males, 1.7% for females) are greater than for those with the maximum pension level (2.5% for males, 1.4% for females). Also for Canada, Wen et al. [31] report a detailed analysis of mortality among Canada and Quebec Pension Plan beneficiaries broken down by pension income (PI) level. Their analysis builds on earlier work by Adam [30]. They conclude that there is significant variation in mortality between all pension levels, especially at younger ages, and that the inequality gap narrows with age.

Fors et al. [18] have tracked income inequality in old-age LE and life span variation in Sweden between 2006 and 2015. Their main result is that the gap in LE at age 65 grew by more than a year between the lowest and highest income quartiles, for both men (from 3.4 years in 2006 to 4.5 years in 2015) and women (from 2.3 to 3.4 years). This widening income gap in old-age LE was driven by different rates of mortality improvement: individuals with higher incomes increased their LE at a faster rate than those with lower incomes.

For Spain, Regidor et al. [39] assess the link between education and occupational class on the one hand and mortality on the other over an 8-year period and seek to identify possible factors to explain it. They conclude that mortality inequalities among older Spanish adults are small. Kulhánová et al. [37], looking to explain this low level of inequality, concluded that smaller inequalities in mortality in Spain were only found for cardiovascular disease and cancer. Inequalities in mortality from most other causes were not smaller in Spain than elsewhere. Permanyer et al. [38] explored the gradient in LE by educational attainment group and inequality in age-at-death distributions within and across those groups for the period between 1960 and 2015 in Spain. Their main conclusion was that health inequality is increasing across education groups and within the least-educated ones.

Solé-Auró et al. [40] computed educational inequalities in LE, healthy life expectancy (HLE) and unhealthy life expectancy (ULE) by gender and education level in Spain for 2012. They detected substantial differences in ULE by gender and education, with a higher effect on women and those with low levels of education. However, they also provided new evidence of a possibly lower gradient in mortality and health in Spain. González and Rodríguez-González [41] analyze the evolution of inequality in mortality in Spain during the period 1990–2018. They focused on age-specific mortality and considered inequality across narrowly defined geographical areas, ranked by average SE status. They found that the decreases in mortality seen over these 28 years in Spain were accompanied by reductions in inequality among younger cohorts, although inequality actually increased over the period for older men.

Finally, Pérez-Salamero González et al. [42] examined differences in LE between self-employed (SEP) and paid employee (PEP) workers when they become retirement pensioners, looking at levels of pension income using administrative data from Spanish social security records. They found that LE at 65 is slightly higher for SEP than for PEP retirement pensioners, despite the fact that the average retirement benefit is (much) lower for SEP than for PEP workers.

To summarize, this section has highlighted the importance of the link between mortality and (pension) income level and the fact that very little is known on this subject in Spain. The present paper helps to fill a gap in the literature because it enriches some of the results presented in Pérez-Salamero González et al. [42] in various ways: introducing the concept of relative mortality, focusing on paid employee (PEP) workers when they become retirement pensioners (self-employed workers when they become retirement pensioners are excluded given the weak link between pension income and LE), and refining the procedure for obtaining life expectancies within groups and including total LE by PI level at age 75.

## Methods

We use the Continuous Sample of Working Lives (CSWL), a Spanish large administrative dataset, that offers several advantages over survey data [46]. CSWL is a random sample of around 1.2 million people, i.e. 4% of the reference population. It contains administrative data on the working lives which provide the basis for the sample taken from Spanish Social Security records and comprises anonymized microdata with detailed information on individuals [47, 48].

The first wave covers people who had a financial link with the Social Security system in 2004 and provides the entire working history of the sample population. The sample is updated every year using information from the variables selected from the Social Security system dating back to when computerized records began, and from other administrative data sources which record additional information on individuals. The data available to researchers runs from 2005 to 2018.

The sample reference population is defined as individuals who have had some connection (through contributions, pensions or unemployment benefits) to the Social Security system at any time during the year of reference. Individuals who for any reason have no connection to Social Security in a particular year do not appear in the CSWL. Nor are public employees included.

Table 1 shows the participants/records excluded (absolute values and percentage) from the initial number of beneficiary records classified according to group selection (G) and technical reasons (T).

**Table 1:** Participants/records excluded from the initial number of beneficiary records classified according to group selection (G) and technical reasons (T)

Items	2005–2010	%	2011–2014	%	2015–2018	%
**Beneficiaries’ records (initial)**	2,132,383	100	1,891,168	100	1,993,204	100
**Invalid date of birth (T)**	− 1686	− 0.08	− 858	− 0.05	− 636	− 0.03
**Female records (G)**	−1,082,974	−50.79	− 962,393	− 50.89	− 1,017,930	− 51.07
**Non-retirement records (G)**	− 413,448	− 19.39	− 357,805	− 18.92	− 360,900	− 18.11
**Administrative errors (T)**	− 4203	− 0.20	− 3140	− 0.17	− 2602	−0.13
**Benefits of deceased pensioners (T)**	−25,209	− 1.18	−22,272	− 1.18	− 24,260	−1.22
**Retirement beneficiaries’ records**	604,863	28.37	544,700	28.80	586,876	29.44
**Early retirement (G)**	− 281,620	−13.21	− 241,433	− 12.77	− 257,241	− 12.91
**Special schemes (G)**	− 147,242	−6.91	−91,516	−4.84	− 107,151	−5.38
**Other excluded records (T)**	− 2790	−0.13	−21,745	−1.15	− 952	− 0.05
**Beneficiaries’ records (final)**	173,211	8.12	190,006	10.05	221,532	11.11

Source: Own work based on [49]

The contributory system in Spain is structured in different “regimes” or schemes, each of which covers a group of workers of a particular type. The General Regime is the essential core of the whole system and includes all employees over 16 not included in another “special system” [42].

In our study the initial population are the (true) retirement pensioners whose retirement age was 65 (the ordinary retirement age) or more within the general scheme. Until 31 December 2012, the statutory retirement age in Spain was 65. From 2027 onwards there will be two standard retirement ages: 65 with 38.5 years’ contributions and 67 with 37 years’ contributions. The shift from 65 to 67 is being made gradually between 2013 and 2027.

Because of lower labor force participation rates among the equivalent female cohorts in Spain and the fact that women sometimes have shorter careers (in terms of years of employment) and may work less intensively than men due to family roles and commitments, females are excluded. Most OECD countries have seen a considerable rise in female labor force participation since the mid-twentieth century. However, Spain lagged behind and until the beginning of the twenty-first century the male breadwinner model was predominant among Spanish families. Lozano and Renteria [50] point out that, among females, the growth in precarious LMLE – labor market LE, which commonly describes the number of years that individuals are expected to be economically active – may indicate that their entry into the labor market over the last 30 years has involved low-quality employment conditions.

Bearing the above reasons in mind, the PI level cannot really be considered a suitable indicator for women’s working-life income in Spain, at least not as far as the data we use are concerned. This is also true for retired German women [34]. In Poland, the impact of pension income on LE is much stronger among men than women, for whom LE is strongly influenced by other factors [51]. The focus of our analysis in this research is therefore the male mortality gradient by pension income.

Mortality among disabled people is far higher than among the general population [52–54], so combining the two populations (retirement pensioners and disabled pensioners) could have a seriously misleading effect when it comes to accurately determining the male mortality gradient by pension income.

Given that poor health is an important reason for early retirement [55], pensioners who access benefits before the statutory retirement age are also excluded. However, it must be said that very little research has explored the impact of early retirement, and the literature highlights the existence of heterogeneous effects, mainly according to occupation and gender. The effects are rarely significant for women [56].

Retirement pensioners belonging to the special system for the self-employed (SEP) are also excluded because the pension rules make their benefits a poor proxy for lifetime income [42]. It is paradoxical that the average retirement benefit for all the pensions in payment was around €10,196 per year in 2018 for the SEP regime. The same figure for the general regime was €17,291 per year in 2018, i.e. 70% higher than in the SEP regime.

Finally, as shown in Table 1, of all the initial beneficiary records for each of the periods considered, by the end – once the whole process of sifting and excluding had been completed – only 8.12% of the original records remain for the first period, along with 10.05% for the second and 11.11% for the third. By far the most important reason for excluding beneficiary records involves group selection.

### Variables and socioeconomic groups

The following variables are available in the CSWL data: Month and year in which the pension was first paid and ended (if ended), regulating base used to calculate the amount of the benefit, years contributed under each pension regime, benefit type (old age pension, early retirement, disability insurance, survivor’s benefits, other), and gender. The design of the Spanish pension system, to a large extent, guarantees that retirement benefits are closely related to lifetime earnings, i.e. the initial pension amount is a (good) proxy for the income stream the pensioners had during their active lives (or at least over the 15–25 years immediately preceding retirement).

Measuring income is not as simple as it may sound. It can be conceptualized at the individual or household level, with the former better reflecting an individual’s earning ability and the latter better capturing living standards [57].

There are basically two main approaches to measuring income: to divide individuals into specific income percentiles, i.e. distribute them into equally sized ordinal groups, or to categorize them into absolute income brackets. Given the features of the database used, we have divided pensioners on the basis of income cut-off points deriving from the pension income distribution of the total population of retirement pensioners. This approach is often used in actuarial and social insurance studies [23, 29–31, 58–60], given that the research outcomes are easier to convey to policymakers and non-technical interested readers.

Table 2 shows the exposures in person-years and number of deaths (percentages) for the “hypothetical pensioner income levels” and periods studied. To analyze the data, we group the records into four PI levels (*B*_*m*_): “1-Low”; “2-Medium-Low”, “3-Medium-High” and “4-High”. We assign pensioners to each group according to the minimum (Min) and maximum (Max) benefits in force at the time of their retirement. The hypothetical scaled pensioners are classified as “Low”, “Medium-Low”, “Medium-High”, and “High” recipients; Low: *B*_*1*_ ≤ Min; Medium-Low: Min < *B*_*2*_ ≤ (0.5Max + 0.33Min); Medium-High: (0.5Max + 0.33Min) < *B*_*3*_ ≤ 0.75Max; and High: *B*_*4*_ > 0.75Max.

**Table 2:** Pensioners by initial pension income level: exposures in person-years and number of deaths by period

Periods	Items	Groups				
		Low	Med-Low	Med-High	High	Total
**2005–2010**	Exposures	22,146	70,622	26,169	28,165	147,102
	% Exposures	15.05	48.01	17.79	19.15	100
	Deaths	1132	3379	937	781	6229
	% Deaths	18.17	54.25	15.04	12.54	100
**2011–2014**	Exposures	20,116	74,562	23,958	37,129	155,764
	% Exposures	12.91	47.87	15.38	23.84	100
	Deaths	970	3539	902	874	6285
	% Deaths	15.43	56.31	14.35	13.91	100
**2015–2018**	Exposures	21,562	83,420	25,995	49,921	180,897
	% Exposures	11.92	46.11	14.37	27.60	100
	Deaths	1009	4003	1040	1138	7190
	% Deaths	14.03	55.67	14.46	15.83	100

Source: Own work based on [49]

In short, pensioners are classified into one group or another according to the initial amount of their pension. This is calculated taking their working life into account, i.e., the initial amount is an indicator of their earnings during the years of their active life (or at least over the 15–25 years immediately preceding retirement).

Once a worker is classified as belonging to a particular group, when they retire, they will remain in that group over time. If we were using pension income percentiles, then given that the composition of the sample changes every year due to deaths and new pensioners being admitted as replacements, some individuals could change quartile (going up to the next one or down to the one below) depending on the initial amounts of the new retirees’ pensions. This reassignment of individuals could have an impact on the results [61].

For the last year considered in our study (2018), there was a minimum pension benefit payable from age 65 equal to EUR 657.60 per month for single pensioners and EUR 811.40 per month for pensioners with a dependent spouse (14 payments per year). If the initial retirement benefit determined according to the rules in force falls below the legislated minimum amount, then a complement is added to it. However, there are certain limitations governing receipt of that complement, depending on overall earnings levels. The group of so-called “low” pensioners therefore includes individuals with benefits below the “official” minimum benefit.

The maximum pension was EUR 2617.53 per month in 2018 with 14 payments per year. In practice, a retirement pensioner can receive a higher benefit (up to 26% more) than the “official” maximum benefit due to the maternity supplement and/or additional amounts for delaying retirement beyond the statutory age.

### Methodology

The male mortality gradient by pension income is analyzed in two complementary ways: First we estimate an indicator called “relative mortality”, which we use to compare graduated death rates among retired male beneficiaries by age group and PI level to the annual death rate among retired male beneficiaries for that age group for three different periods: *P*_*1*_: 2005–2010, *P*_*2*_: 2011–2014, and *P*_*3*_: 2015–2018. The first period is 5 years whereas the second and third are 4 years. The actual number of deaths and the risk exposure used to calculate the crude annual death rate come from the CSWL (Tables 1 and 2).

For each age group we calculate relative mortality rates at various PI levels. The final graduated beneficiary mortality rates, ${q}_{x,\mathrm{P}}^{j,m}$, represent the best estimates of the rates for period-year interval P. Once the graduation process has been concluded, the relative mortality ratio, ${RM}_{h,\mathrm{P}}^{j,m}$, can be obtained immediately. This is the ratio between the death rate of a subgroup and the death rate of the group as a whole [23]. For the subgroup of pensioners in age interval *h*, with PI level *m* and gender *j*, for period-year interval *P*, it is:

1 $${RM}_{h,\mathrm{P}}^{j,m}=\frac{q_{h,\mathrm{P}}^{j,m}}{q_{h,\mathrm{P}}^{j,T}}$$

A relative mortality ratio of 1.00 for a PI level indicates that it is the same as the death rate for that age group as a whole. A relative mortality ratio of less than 1.00 means that the rate for that PI level is lower than the death rate for that age group as a whole (or any other group of interest), while a rate of more than 1.00 means that the death rate is higher than for that age group as a whole.

If the death rate is higher for low PI beneficiaries than for high PI beneficiaries but is constant over time, then these mortality differentials by SE status will show no trend over time. But if the death rate for the longer-lived group declines more quickly than for the shorter-lived group, then mortality gaps will widen over time. Conversely, if the reduction in mortality for the shorter-lived group improves faster than for the longer-lived group, then mortality gaps will narrow over time. Mortality gaps could also narrow if the rate of mortality increases for the longer-lived group but declines or remains steady for the shorter-lived group; and gaps could widen if the mortality rate increases for the shorter-lived group but declines or remains steady for the longer-lived group.

The technicalities for calculating the relative mortality risk ratio can be seen in the technical Appendix.

As shown below, if the information provided by the relative mortality ratio is not properly understood it could mask real differences for a given PI group, so an additional indicator needs to be introduced to give a broad picture of the true extent of inequality in mortality [62].

The second way of quantifying the male mortality gradient by income is by estimating changes in total LE by PI level at age 65 (LE_65_) and 75 (LE_75_) over time. More specifically, we use the Mort1Dsmooth function in the MortalitySmooth R package [63], which is tailored for mortality research, to construct complete-period life Tables (LT) from age 65 to age 101 and calculate LE_65_ and LE_75_ for the three periods analyzed. In order to check the robustness of the estimated changes in total LE by PI, we have also used an R Package for Mortality Rates Graduation by Discrete Beta Kernel Techniques [64].

Finally, the technical details for testing whether there is a significant positive difference in LE can be seen in the technical Appendix [65–67].

## Results

Table 3 shows the relative mortality ratios by age group and PI level for the three different periods considered, running from 2005 to 2018: P_1_: 2005–2010, P_2_: 2011–2014, and P_3_: 2015–2018. These ratios are calculated according to formula (1).

**Table 3:** Relative mortality ratios by age group and initial pension income (PI) level

Periods	Age	PI level				Dif.
	Group	Low	Med-Low	Med-High	High	Low-High
**P**_**1**_**: 2005–2010**	65–69	1.49	1.03	0.95	0.77	0.72
	70–74	1.18	1.05	0.86	0.83	0.35
	75–79	1.12	1.04	0.92	0.89	0.23
	80–84	1.05	1.02	0.95	0.92	0.13
	85+	1.00	1.01	0.90	0.90	0.10
	**Total**	**1.18**	**1.13**	**0.86**	**0.61**	**0.57**
**P**_**2**_**: 2011–2014**	65–69	1.59	1.02	0.94	0.85	0.74
	70–74	1.21	1.05	0.89	0.82	0.37
	75–79	1.11	1.00	0.96	0.94	0.17
	80–84	1.06	1.03	0.95	0.84	0.22
	85+	1.05	1.05	0.89	0.81	0.26
	**Total**	**1.21**	**1.15**	**0.80**	**0.59**	**0.62**
**P**_**3**_**: 2015–2018**	65–69	1.67	1.09	0.93	0.75	0.92
	70–74	1.37	1.07	0.91	0.79	0.58
	75–79	1.22	1.07	0.95	0.85	0.37
	80–84	1.07	1.06	0.91	0.86	0.21
	85+	1.05	1.08	0.85	0.83	0.22
	**Total**	**1.25**	**1.19**	**0.78**	**0.58**	**0.67**

Source: Own work based on [49]

For 2005–2010 the relative ratios for the male 65–69 age-group are 1.49, 1.03, 0.95, and 0.77 from the lowest PI level to the highest. The figure of 1.49 for the lowest PI level means that the death rate is 49% higher than for that age group as a whole, while the figure of 0.77 for the highest PI level means that the death rate is 23% lower than for that age group as a whole. Table 2 shows that in groups containing older ages there is less of a difference in relative mortality rates between PI levels.

A comparison of interval P_1_: 2005–2010 with P_2_: 2011–2014 shows a generalized increase in relative mortality inequality for almost all age groups, with the exception of the 75–79 group, where the difference in relative mortality between the lowest and the highest PI levels drops from 0.23 to 0.17, i.e. the relative mortality inequality within this age group is lower than in the previous period.

A comparison of interval P_2_: 2011–2014 with P_3_: 2015–2018 shows a substantial increase in relative mortality inequality for the youngest age groups (65–69; 70–74 and 75–79) and a slight reduction for the rest (80–84; and 85+).

Table 3 also shows the trend in relative mortality ratios for the whole range of age groups and periods analyzed. It reveals that the difference in death rates across PI levels (column Dif. Low-High) widened further for male retirement pensioners. For 2005–2010, the gap in relative mortality rates for the whole range of age groups between pensioners in the lowest and the highest income groups is 0.57. This gap widens over time and reaches 0.67 in 2015–2018. Overall, the literature reviewed [21–36] generally indicates that when mortality gaps have widened over time in the past, the probabilities of death have usually fallen faster for high-status groups than for low-status groups. As can be seen in Table 4, this is what actually happens in the Spanish data studied here.

**Table 4:** Mortality improvements by age group and initial pension income (PI) level

Periods	Age	PI level				Total
	Group	Low	Total	Med-High	High	
**P**_**1**_**-P**_**2**_	65–69	− 3.78%	4.07%	4.34%	−7.67%	3.12%
	70–74	3.15%	5.45%	2.35%	7.52%	6.04%
	75–79	9.47%	11.28%	4.70%	3.43%	8.34%
	80–84	7.46%	7.74%	8.94%	16.11%	8.55%
	85+	−4.22%	−3.90%	0.99%	9.47%	−0.07%
	**Total**	**1.58%**	**1.98%**	**4.31%**	**7.50%**	**4.01%**
**P**_**2**_**-P**_**3**_	65–69	−11.56%	−12.97%	−5.99%	6.37%	−6.64%
	70–74	−8.00%	2.81%	2.82%	8.63%	4.70%
	75–79	−4.63%	−1.60%	6.61%	14.05%	5.23%
	80–84	2.52%	0.83%	7.04%	1.47%	3.39%
	85+	6.61%	3.98%	10.85%	5.31%	6.86%
	**Total**	**1.41%**	**1.79%**	**6.90%**	**7.22%**	**4.95%**
**P**_**1**_**-P**_**3**_	65–69	−15.78%	−8.38%	−1.39%	−0.81%	− 3.31%
	70–74	−4.60%	8.10%	5.11%	15.50%	10.45%
	75–79	5.29%	9.86%	11.00%	16.99%	13.13%
	80–84	9.79%	8.50%	15.35%	17.34%	11.65%
	85+	2.67%	0.24%	11.73%	14.27%	6.80%
	**Total**	**2.97%**	**3.73%**	**10.91%**	**14.18%**	**8.76%**

Source: Own work based on [49]

Table 4 shows the mortality improvements (%) by age group and pension income (PI) level. These figures are obtained by comparing the graduated death rates by age weighted by the number exposed to risk for each of the periods.

Obviously, when the mortality improvement for a given age group has a negative value, for example −3.78% for period P_1_-P_2_ and age group 65–69, the real meaning is that there is a mortality deterioration, i.e. the death rates are higher in this period (P_2_) than in the previous one (P_1_) for this age group.

The reduction in death rates measured by PI levels over the two intervals (P_1_-P_3_) fully explains the change in relative mortality inequality. This reduction is (much) higher for the “High” (14.18%) and the “Medium-High” groups (10.91%) than for the whole group (8.76%) and given that the previous relative mortality rates were less than 1.00 for both groups (0.61 and 0.86 respectively in period 1), the figures for the rates of these two groups in the third period (0.58 and 0.78 respectively) indicate that inequality in mortality has increased over time. For the case of the “Low” and “Medium-Low” groups the improvements in mortality are (much) lower (2.97 and 3.73% respectively) than for the whole group (8.76%), so the figures for their ratios in this second period (1.25 and 1.19 respectively) are further from 1.00 than in the first period (1.18 and 1.13 respectively).

For the whole period considered the improvement in mortality is quite substantial at 8.76% for the group of pensioners as a whole but varies widely from one pensioner income level to another. The higher the PI level, the greater the improvement in mortality.

It is worth noting that the improvement in mortality is not uniform across age groups. If the results are observed over two intervals (P_1_-P_3_), then for some age groups there has been a deterioration in mortality (the age group 65–69 in all the PI levels, and the age group 70–74 in the case of the “Low” group). If we look at the result for the age groups without considering the PI level, i.e. the last column in Table 4, the age group 65–69 is the one that has suffered a deterioration in mortality (− 3.31%), with the age group 75–79 being the one with the most notable improvement in mortality (13.13%).

At first glance, it might appear that the observed relative mortality ratios found for the periods analyzed would imply enormous differences in LE between the groups of beneficiaries sorted by PI levels. However, as shown below, this is not entirely the case.

**Fig. 1:**
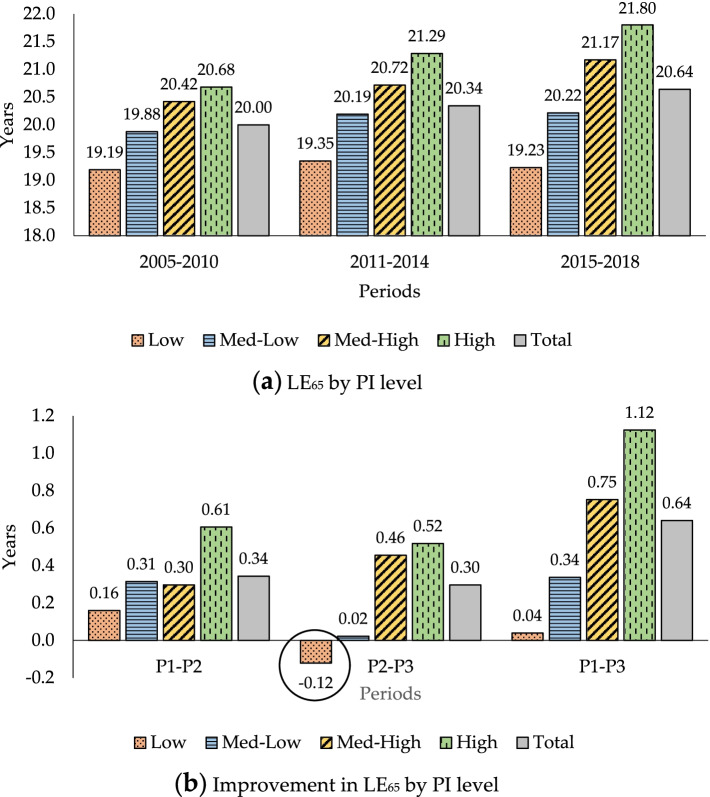
LE_65_ and improvement in it measured by initial pension income (PI) level. **a** LE_65_ by PI level. **b** Improvement in LE_65_ by PI level

Figure 1 shows complete LE at age 65, LE_65_, and the improvements in it measured by PI level for the periods studied. This figure is broken down into 2 graphs.

Graph (a) in Fig. 1 shows that LE_65_ has a positive link with PI level. The higher the PI level, the higher the LE at age 65. Graph (b) shows that across all SE groups (total), LE_65_ increases by 0.34 years between the first and second periods and by 0.30 years between the second and third. For the same periods shown for Graph (a) in Fig. 1, the LE_65_ of the general population (males) increased by 0.81 and 0.30 years respectively [68]. In the first period, the absolute mortality improvements are largest in the most advantaged group (0.61 years) and smallest in the most disadvantaged group (0.16) For the second period, beneficiaries in the most advantaged group also show a mortality improvement of 0.52 years while those in the most disadvantaged group show a small decline in their LE (− 0.12 years). For the whole period (P_1_-P_3_), it can be said that the higher the PI level, the greater the improvement in LE.

**Fig. 2:**
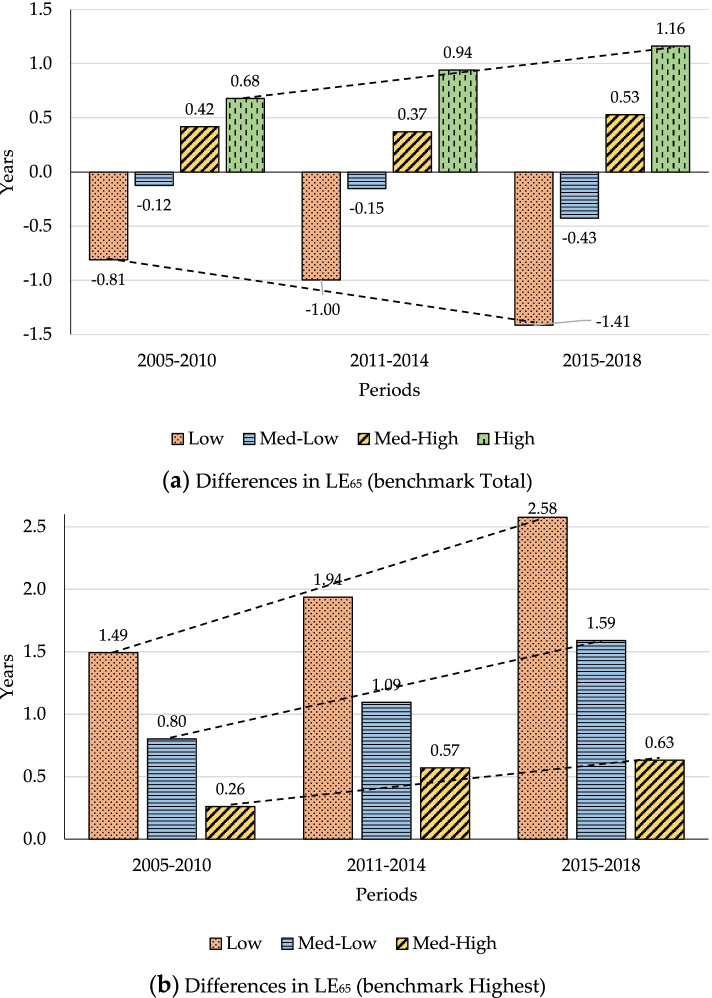
LE_65_ and differences in it by initial pension income (PI) levels. **a** Differences in LE_65_ (benchmark Total). **b** Differences in LE_65_ (benchmark Highest)

Figure 2 shows LE_65_ and its absolute differences by PI levels for the periods studied. This figure is also broken down into 2 graphs: Graph (a) shows the difference in LE_65_ between each group and the total LE while Graph (b) shows the difference in years using the highest PI group as a benchmark.

The results are remarkable: Absolute differences between the highest and lowest groups are found to have widened over time. For both groups in the third period, LE is further from that of the population as a whole than in the first period (Graph (a)). Graph (b) provides valuable information: For 2005–2010, a gap of 1.49 years between pensioners in the lowest and the highest income groups is reported. This gap widens over time and reaches 2.58 years in 2015–2018. A similar trend can be observed if the highest PI group is compared with the other two groups. The linear trend lines point to a constant increase in SE mortality disparities for all groups, but with a steeper social gradient in the lowest PI group.

**Fig. 3:**
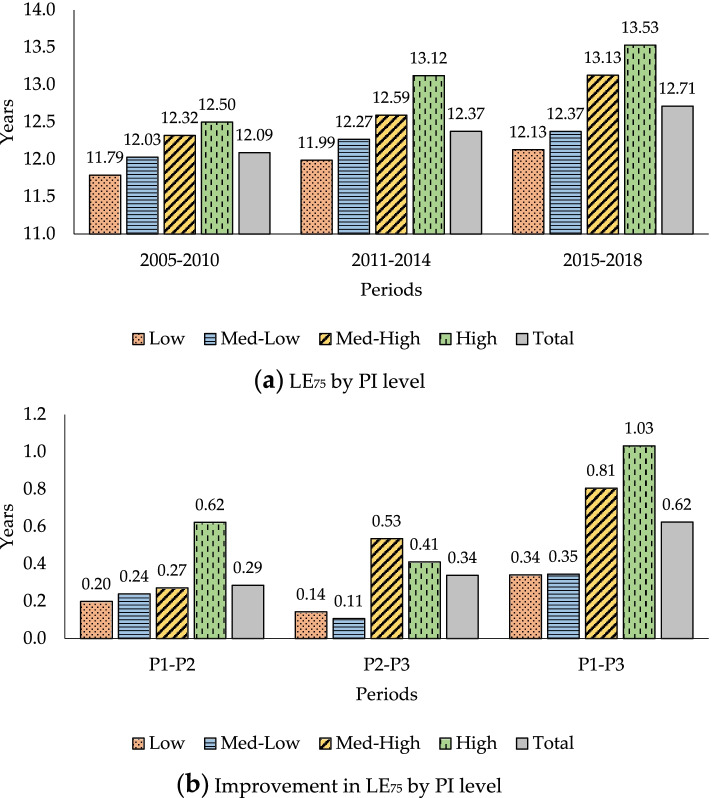
LE_75_ and differences in it by initial pension income (PI) levels. **a** LE_75_ by PI level. **b** Improvement in LE_75_ by PI level

But what about LE at older ages? Figure 3 shows complete LE at age 75, LE_75_, and the improvement in it by PI levels for the periods under study. This figure is also broken down into 2 graphs.

Similar comments to those on the previous figure can be made about LE_75_ and improvement in it by PI levels for the periods studied. For the whole period (P_1_-P_3_) it can be said that the higher the PI level, the greater the improvement in LE. However, the second period (P_2_-P_3_) is an exception: LE_75_ gain is larger for the lowest group (0.14 years) for the Medium-Low group, and the same finding emerges when the Medium-High group (0.53 years) is compared to the most advantaged group (0.41). The rationale behind this is the observed fact (Table 4) that the rate of mortality improvement is not uniform across the age range within each PI level, i.e. the age-death rate structure for each group of beneficiaries by PI levels does not changes proportionally over time. The changing gaps in LE between several PI groups depends on differential changes in age-specific mortality rates and differences in “survivability”, a summary measure of initial age-specific mortality rates. Survivability at a given age measures the likelihood of surviving until this age multiplied by the expected remaining life years after surviving to this age. Intuitively, a person only benefits from a reduction in an age-specific mortality rate if they have survived until this age (ex ante effect) and, if so, the benefit is the expected extra life years thereafter (ex post effect) [62].

It is worth highlighting that the results shown in Figs. 2 and 3 are almost identical using the R Package for Mortality Rates Graduation by Discrete Beta Kernel Techniques [64].

These results in Figs. 2 and 3 raise the question as to whether LE_65_ and LE_75_ differences between PI levels are statistically significant or not.

Table 5 shows us more detailed information. For all three periods analyzed, it shows the differences in LE_65_ and LE_75_ (DLE_65_ and DLE_75_, respectively) from one PI group to another, from Med-Low and Low to High and Med-High, together with the standard error for those differences and the z-score value of the test statistic to test the null hypothesis that the difference in LE is zero against the alternative of its being positive.

**Table 5:**
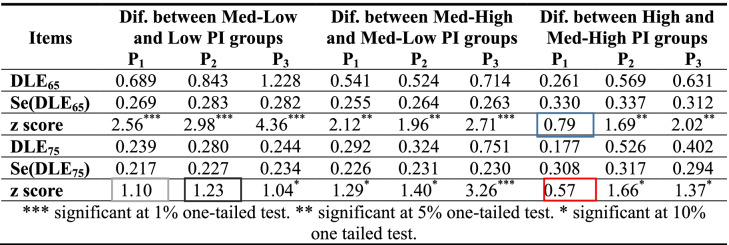
Absolute differences in LE between initial pension income (PI) groups by periods and ages

*** significant at 1% one-tailed test. ** significant at 5% one-tailed test. * significant at 10% one tailed test

Source: Own work

The results show that most DLE_65_ are statistically significant at 1% or 5%, with the sole exception of the difference between High and Med-High PI groups in 2005–2010, which is not significant at 10%. Apart from the same exception as in the case of LE_65_, differences in LE_75_ between Med-Low and Low PI groups are not statistically significant. For the rest of the comparisons, some are statistically significant at 1% and others at 5% or 10%. These results thus support the idea that there is highly significant evidence of a positive relationship between LE_65_ and PI groups. As expected, given that at older ages there is less of a difference in relative mortality ratios between PI levels, the statistical significance diminishes slightly as the figures rise to age 75 and older.

What is shown in the above table can be seen more intuitively in Table 6, which shows 95% confidence intervals (CIs) LE_65_ and LE_75_ for all PI groups of pensioners and all three periods analyzed.

**Table 6:**
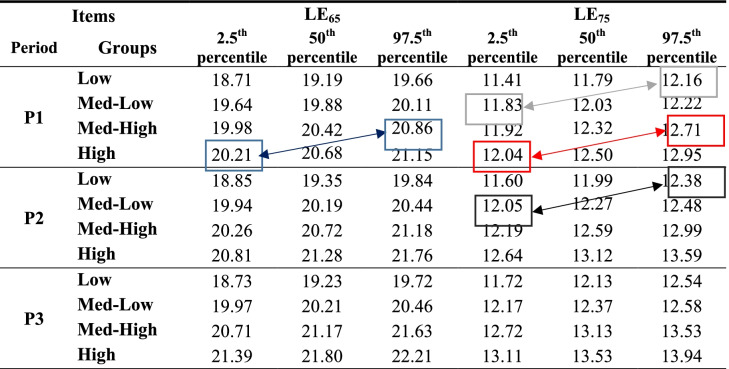
LE_65_ and LE_75_. 95% Confidence intervals by initial pension income (PI) groups and periods

Source: Own work

As Table 5 indicates, for the first period and the case of LE_65_, the difference between high and Med-High PI groups is not statistically significant. The intuitive reason can be seen in Table 6; the upper bound of 95% CI (97.5th percentile) for the Med-High group is higher (20.86 years) than the lower bound of 95% CI (2.5th percentile) for the best-off group of pensioners (20.21 years). For the case of LE_75_ the same intuitive explanation applies. The upper bound of 95% CI for the Med-High group is higher (12.71 years) than the lower bound of 95% CI for the best-off group of pensioners (12.04 years). As a general rule, the greater the difference between the upper bound of a PI group “m” and the lower bound of an adjacent (wealthier) PI group, the more likely it is that the null hypothesis that the difference in LE is zero will be accepted.

**Fig. 4:**
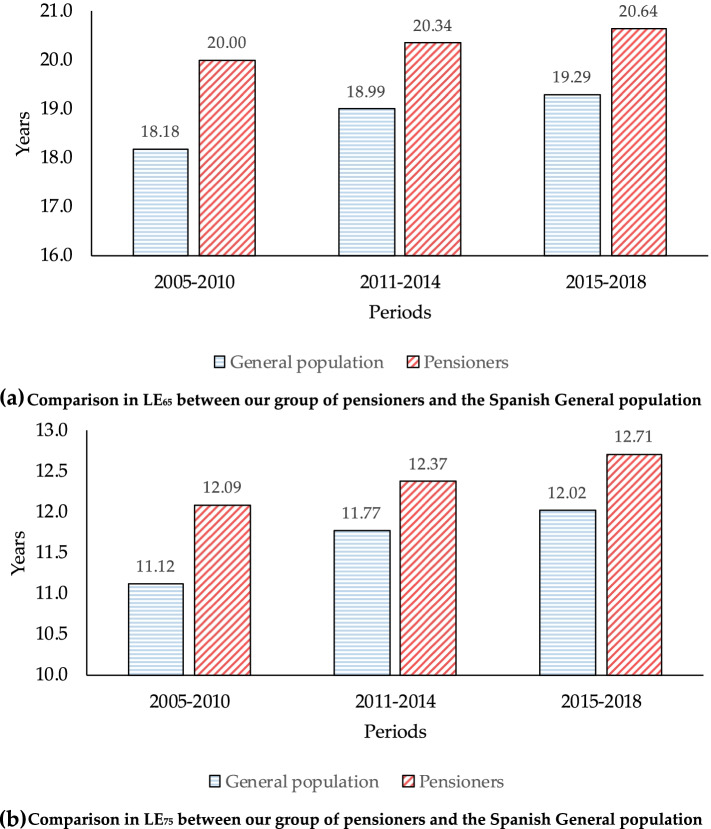
LE_65_ and LE_75_. Comparison between our group of pensioners and the Spanish general population. **a** Comparison in LE_65_ between our group of pensioners and the Spanish general population. **b** Comparison in LE_75_ between our group of pensioners and the Spanish general population

We also present a comparison of our results with the LE of the Spanish population as a whole [68] (Fig. 4, Graph (a) for LE_65_ and Graph (b) for LE_75_).

As expected, individuals within the sample live longer than the general population because one of the requirements for obtaining a retirement pension is to have contributed for at least 15 years, including at least 2 of the last 15 years. This requirement is expected to exclude some of the most at-risk members of the Spanish population because of the strong correlation between labor force participation and health observed in several countries [24, 69–71]. It is worth recalling that disabled beneficiaries and early retirees, collectives with lower LE than general population, were also excluded.

What is remarkable is the fact that LE_65_ for pensioners is around 10% higher (1.82 years) than for the general population in the first period, but this relative advantage narrows over time; for the third period it is only around 7% (1.35 years). Unsurprisingly, the relative difference in LE_75_ compared with the general population is smaller than for LE_65_. For the first period, LE_75_ for pensioners is around 8.72% higher (0.97 years) than for the general population, whereas for the third period it is only around 5.74% (0.69 years).

## Discussion

We find an inverse relationship between PI levels and mortality for male retirement pensioners. The trend over the full period analyzed shows that the difference in LE by PI level has widened. Although it should be considered with due caution, given that the selection of pensioner groups to be studied are not completely equivalent for the various countries with similar research based on pension fund and Social Security data, our result is robust and in line with studies on Germany [32–34] and the USA [15, 24], which have also reported increasing inequalities among elderly men. Similarly, to what has been reported for other countries, at older ages the differences in death rates (LE) across pension levels diminish. Researchers frequently use the “age-as-leveler” hypothesis to explain decreasing inequality and a weakened relationship between income position and mortality in advanced old ages (from 80 to 85 onwards) [72].

Likewise, two recent investigations for Chile [58] and Argentina [59], also based on Social Security records, report differences in LE_65_ inequality among retired men by pension income level very similar to our findings. In Chile it is reported that there is a three-year difference in LE_65_ between the lowest and highest income groups in both men and women, whereas for Argentina the difference is around 2.5 years.

Our findings also reveal that, in a European context, LE inequality among retired Spanish men is relatively small. This is in line with previous findings for Spain involving older adults and using very different methodologies and/or databases [37–41]. Regidor et al. [39], for example, conclude that mortality inequalities in older Spanish adults are small. The ubiquity of social safety nets and widespread adherence to the Mediterranean diet may be responsible for this finding. The research by Kulhánová et al. [37] suggests that these smaller inequalities in mortality seem to be a historical coincidence rather than the outcome of deliberate policies. Mackenbach et al. [73] also report that relative inequalities in mortality are largest in the East (Czech Republic, Lithuania, Hungary, Estonia, and Slovenia) and smallest in the South (Spain and Italy). Finally, Solé-Auró et al. [40] suggest that this lower gradient in mortality may in part be explained by a later process of economic modernization. It might also be explained by the existence of health assets that have traditionally received less attention in these countries. These assets, such as greater family network density and less inequality of access to healthy food, can be critical elements in political action.

Given the data used in this paper (Spanish Social Security data have limited socioeconomic information), providing a coherent explanation of why LE inequalities are small is no easy task. Along with other behavioral and structural factors [73], a combination of factors such as the design of the pension system, the universality and good quality of the health system, and high levels of family support could explain why LE inequalities for retired Spanish men are relatively small.

The Spanish pension system plays an important role in maintaining quality of life, especially in the fight against poverty for older persons [74, 75]. Spain has one of the highest aggregate replacement ratios in Europe. Spanish pensioners largely maintained their relative standard of living during the recent economic crisis (2008–2014). The percentage of people in Spain aged 65 and over whose income is lower than 50% of median equalized household disposable income is lower (9.4) than the figure for the total population (15.5). This rate is also 4.1 percentage points below the average for OECD countries (13.5%) in 2016 [76].

The contributory retirement system has been shifting from an insurance-based system to an increasingly redistributive system; the minimum retirement benefit for a single pensioner at age 65 increased by 15.24% in real terms from 2000 to 2018 and the maximum benefit decreased by 5.86% in real terms. The ratio between maximum and minimum pensions has greatly decreased over time, from 5.07 (2000) to 4.14 (2018). Around 91% of people aged 65 and over live in owner-occupied homes, and only 1.8% were found to be living in overcrowded households [74].

In the study of multidimensional comparison of countries’ adaptation to societal aging [77], Spain is the highest ranked in security, a major component of the multidimensional Aging Society Index that assesses the status of older populations. The component income includes five items: income, pension wealth, public expenditure on long-term care, government debt, and physical safety.

The Spanish health system is based on the principles of universality, free access, equity, and fairness of financing, and is mainly funded by taxes. Spain has the most efficient healthcare system in Europe. In the Healthcare Access and Quality Index, Spain is ranked 19th of 195 countries for healthcare and access [78].

Even though healthcare is universally guaranteed and free at point of use, inequalities in access to adequate healthcare still exist, especially in rural areas. As reported for Italy by Federico et al. [79], in Spain family continues to be the main source of support and help in old age; family support and informal social safety nets, especially in the many low and medium income families in the country, may have mitigated reported inequalities in access to (universal and state-organized) healthcare services.

In Spain, it is common for older people to live in their own homes until they are left alone by the death of their partner, fall ill and/or suffer disability and then for them to reside with a family member, usually a daughter [80]. Family life is characterized by a high frequency of personal contact between generations. The intensity of family contacts provides a basis for the flow of mutual support between members of the family network.

A more recent study [81], based on the Spanish sample of the Survey of Health, Aging and Retirement in Europe (waves 2006 and 2013), concludes that families in Spain remain highly involved in elderly care.

As stated in the previous section, the gap in LE widens over time, from 1.49 to 2.54 years and from 0.71 to 1.40 years respectively for pensioners aged 65 and 75, and these differences are statistically significant. Perhaps one reason for the increase in inequality in LE observed in this paper can be found in the lack of investment in the public health system. Over the period 2008–2013, annual per-capita spending on health (in real terms) decreased by − 1.9%, better than only Greece, Portugal and Iceland of the OECD36 countries [82]. Growth during the period 2013–2018 was positive at 2.3%, very close to the average for the period for all the 36 countries of the OECD studied (2.4%).

Similarly, the macroeconomic figures for total spending on health fell from 9.4% of GDP in 2009 to 9.1% in 2018, but the variation in the proportions of public and private spending was very different [82]. This could partly explain the increase in inequality, since the SE groups with bigger pensions would presumably benefit most from these private health services. Public spending on health fell from 7.1% of GDP in 2009 to 6.4% in 2018, while spending on the private health sector rose from 2.3 to 2.7% over the same period.

Another possible explanation for the growth of inequalities in LE_65_ could be the fact that there has been a shift in inequalities from younger to older ages in Spain [41]. This is considered a survival effect [36]. What is certain is that, as pointed out by Mackenbach [83], health inequalities are influenced in sometimes unexpected ways by factors that are beyond our control.

In order to establish the reasons behind this increased inequality in LE, more research needs to be carried out [84]. An analysis of all Spanish social security records instead of just a sample could shed some light on the matter.

### Limitations

To conclude this section, some limitations to the study should be taken into account.

First, we are aware that pension income is not a perfect indicator of a beneficiary’s total income [58]; other forms of income could arise from partial employment after retirement, return on investments and savings, government and private transfers, etc. … However, it is worth noting that 70% of retirement pensioners in Spain have only their public pension as a source of income. Therefore only 30% of retirement beneficiaries have additional income from private pensions and savings, insurance plans, financial products and rentals [85]. It could be said that for pensioners with lower benefits, the amount of the retirement pension is a good indicator of their total income, although for pensioners with higher benefits, this amount may not be such a good proxy of their total income.

Second, we excluded groups of pensioners for whom the application of our SE indicator might not have been suitable for various reasons (disabled pensioners, early retirees, beneficiaries in special schemes such as the self-employed).

Third, due to the fact that they are not included in the database used, we were unable to work with the collective of pensioners belonging to the Régimen de Clases Pasivas (civil servants).

Fourth, the sample selection we used does not allow us to draw conclusions about the whole population, and not even about all retirement pensioners, but it does work for a large part of this collective, since those that retired from the general regime in 2018 represent 73.88% of the total number of retirement pensioners [86].

Finally, despite the fact that the results we have obtained are coherent, the analysis could be carried out in much greater depth if we had access to all the records held by the Department of Social Security, along with details of any additional sources of income the pensioners may have.

## Conclusions and future research

We have found an inverse relationship between PI levels and mortality for a selected group of male retirement pensioners (first research question). Given that we selected a representative sample from the total population of male retirement pensioners [47, 48], it can be said that the weight within the Spanish public pension system of the beneficiaries analyzed in this study is by no means negligible: the amount of pension expenditure for this group represented 81.27% of the total expenditure on retirement pensions for men in 2018.

We have also found highly significant evidence of a positive relationship between LE_65_ and pension income (second research question), and that the trends for the entire period analyzed show that the gap in LE as measured by PI levels has widened over time (third research question).

We have also shown that, if the information provided by the relative mortality ratio is not properly understood, then it could mask real differences in LE for a given PI group. A second indicator therefore needs to be introduced to give a broad picture of the true extent of inequality in mortality.

Our findings show that inequalities in mortality for retirement pensioners are small but slightly higher than previously reported for Spain [42]. The differences can be mainly explained by the exclusion of SEP retirement pensioners, by improvements made to the procedure for obtaining life expectancies within groups, and by some additional adjustments made in the dataset used. This is in line with previous findings for Spain involving older adults and using very different methodologies and/or databases [37–41].

The increased inequality in LE does not appear to stem from the pension system reforms carried out over the period 2011–2013, given that the Spanish system has become more redistributive, and the amount of the minimum pensions has clearly increased in real terms over recent years. The causes might be found either in the decreased spending on public health during the period 2009–2018 and the increased spending on private health, which would presumably be of greater benefit to those pensioners with higher incomes, or in the fact that there has been a shift in inequalities from younger to older ages in Spain. To establish the reasons behind this increased inequality in LE, more research needs to be carried out. An analysis of all Spanish social security records instead of just a sample could shed some light on the matter.

As regards the (informal) government proposal to take into account longevity by income (average earnings) status to calculate the initial retirement pension [43], this does not seem to be a very good idea for several reasons (fourth research question): (i) the pension system has been shifting from an insurance-based system to an increasingly redistributive scheme, (ii) unlike the case of the USA [25, 44], SE differences in mortality are much smaller in Spain, and (iii) given that the system does not distinguish by gender when determining the initial amount, we would need to know whether the differences found for retired men by pension income level also occur for women.

Finally, based on the data used and the methodology applied in this paper, one direction for future research would be to check the robustness of our findings. Would they be the same if, instead of a small sample of pensioners, we had access to all the records held by the Department of Social Security along with details of any additional sources of income the pensioners may have?

## Data Availability

Ethics approval is not required to use CSWL; its use for scientific purposes is regulated since inception. Researchers can request versions of the CSWL by post. A separate request must be made for each version. Requests consist of a user profile describing the project being carried out and a document accepting the CSWL’s conditions of use. These are available at the following address: http://www.seg-social.es/wps/portal/wss/internet/EstadisticasPresupuestosEstudios/Estadisticas/EST211

## Appendix

### Technical Appendix

For all beneficiary groups classified by PI level *m*, the crude mortality rate for a given period-year interval, P = {*a*, *a* + 1, ⋯, *n*}, age *x*, and sex *j*, is defined as the observed probability that a person of age *x* nearest birthday will die between ages *x* and *x* + 1 during the period-year interval *P*. *n* represents 31st December for the last calendar year within the period and *a* represents 31st December for the first year.

The observed probability of death is calculated by simply dividing the relevant number of deaths (${D}_{x,\mathrm{P}}^{j,m}$) by the number of life-years of exposure over the given year or period (${E}_{x,\mathrm{P}}^{j,m}$).

The size of the exposure population is estimated by averaging the population sizes at the beginning and end of the year. In our case, the crude mortality rate ${\hat{q}}_{x,\mathrm{P}}^{j,m}$ is calculated as follows:

2 $${\hat{q}}_{x,\mathrm{P}}^{j,m}=\frac{D_{x,\mathrm{P}}^{j,m}}{E_{x,\mathrm{P}}^{j,m}}=\frac{D_{x,a+1}^{j,m}+\dots +{D}_{x,n}^{j,m}}{\frac{1}{2}\left({D}_{x,a+1}^{j,m}+..+{D}_{x,n}^{j,m}+{L}_{x,a}^{j,m}+{L}_{x,n}^{j,m}\right)+{L}_{x,a+1}^{j,m}..+{L}_{x,n-1}^{j,m}}$$

where ${D}_{x,\mathrm{t}}^{j,m}$ is the observed number of deaths of individuals who have attained age x on their nearest birthday for PI level group m, gender *j* in calendar year *t* ∈ {*a* + 1, ⋯, *n*}, and ${L}_{x,\mathrm{t}}^{j,m}$, with *t* ∈ {*a*, …. *n*}, is the observed number of retirement pensioners aged *x* at their nearest birthday in PI level group *m* and gender *j*, at the end of year *t* ∈ *P*.

The average crude death rate ${\hat{q}}_{h,\mathrm{P}}^{j,m}$ for age group *h*, PI level *m*, gender *j* for period-year interval P can be expressed as:

3 $${\hat{q}}_{h,\mathrm{P}}^{j,m}=\frac{\overset{D_{h,\mathrm{P}}^{j,m}}{\overbrace{D_{h,a+1}^{j,m}+\dots +{D}_{h,n}^{j,m}}}}{\underset{E_{h,\mathrm{P}}^{j,m}}{\underbrace{\frac{1}{2}\left({D}_{h,a+1}^{j,m}+\dots +{D}_{h,n}^{j,m}+{L}_{x,a}^{j,m}+{L}_{x,n}^{j,m}\right)+{L}_{x,a+1}^{j,m}+.\dots +{L}_{x,n-1}^{j,m}}}}$$

where ${D}_{h,t}^{j,m}$ is the observed number of deaths for age group P, PI level *m*, gender *j* in calendar year *t*, and ${L}_{h,t}^{j,m}$ is the registered number of retirement pensioners in age group *h*, PI level *m*, gender *j*, at the end of year *t* ∈ *P*.

It is straightforward to see that the average crude death rate can also be calculated as a weighted average of the crude death rates for the beneficiaries with ages included in age group *h*, with the weighting being the number of pensioners-years exposed to risk of death:

4 $${\hat{q}}_{h,\mathrm{P}}^{j,m}=\frac{\sum_{\forall x\in h}{\hat{q}}_{x,\mathrm{P}}^{j,m}\bullet {E}_{x,\mathrm{P}}^{j,m}}{\sum_{\forall x\in h}{E}_{x,\mathrm{P}}^{j,m}}=\frac{\sum_{\forall x\in h}{\hat{q}}_{x,\mathrm{P}}^{j,m}\bullet {E}_{x,\mathrm{P}}^{j,m}}{E_{h,\mathrm{P}}^{j,m}}$$

where ${E}_{h,\mathrm{P}}^{j,m}$ is the number of pensioners exposed to risk included in age group *h*, PI level *m*, gender *j*, for the period-year interval P.

Similarly, if all PI levels are considered the average crude death rate ${\hat{q}}_{h,\mathrm{P}}^{j,T}$ for the pensioners in age group *h*, sex *j* for the period-year interval P can be expressed as:

5 $${\hat{q}}_{h,\mathrm{P}}^{j,T}=\frac{\sum_{m=1}^s{\hat{q}}_{h,\mathrm{P}}^{j,m}\bullet {E}_{h,\mathrm{P}}^{j,m}}{\sum_{m=1}^s{E}_{h,\mathrm{P}}^{j,m}}=\frac{\sum_{m=1}^s{\hat{q}}_{h,\mathrm{P}}^{j,m}\bullet {E}_{h,\mathrm{P}}^{j,m}}{E_{h,\mathrm{P}}^{j,T}}$$

where ${E}_{h,\mathrm{P}}^{j,T}$ is the number of beneficiaries exposed to risk included in age group *h*, at any PI level *m*, gender *j*, for the period-year interval *P*.

Given that the levels of exposure are not sufficiently high for some age groups; the initial estimates must be revised to produce smoother estimates (graduated mortality rates) using a procedure called graduation. In our case the average crude death rates are graduated through the age, PI level, and period dimensions to reflect a compromise between smoothness and fit.

Finally, testing whether there is a significant positive difference in LE $L{E}_{x,P}^m$ between two pensioner income groups for a given age and period, one with a higher PI than the other, can be implemented using one-tailed statistical tests based on normal distribution [65–67]. Under the null hypothesis, this difference will be zero and under the alternative it will be positive.

The z score statistic is defined as the ratio of $DL{E}_{x,P}^{m_i-{m}_j},$the difference in LE between the groups, to the standard error of that difference, $SDL{E}_{x,P}^{m_i-{m}_j}$ which is computed as the square root of the sum of the variances of the corresponding $L{E}_{x,P}^m$for each group, $VL{E}_{x,P}^{m_i}$and $VL{E}_{x,P}^{m_j}$ (respectively)

6 $${z}_{x,P}^{m_i-{m}_j}=\frac{DL{E}_{x,P}^{m_i-{m}_j}}{SDL{E}_{x,P}^{m_i-{m}_j}}=\frac{DL{E}_{x,P}^{m_i-{m}_j}}{\sqrt{VL{E}_{x,P}^{m_i}+ VL{E}_{x,P}^{m_j}}}$$

Following Chiang [65] the variance of the corresponding LE at age *x* for a given group m and period P can be calculated as,

7 $$VL{E}_{x,P}^m=\sum_{k=0}^{w-1-x}\left[{\left({}_k{p}_{x,P}^m\right)}^2\bullet {\left({LE}_{x+k+1,P}^m\right)}^2\bullet \frac{{\left({q}_{x+k,P}^m\right)}^2\bullet \left(1-{q}_{x+k,P}^m\right)}{D_{x+k,P}^m}\right]$$

where ${}_k{p}_{x,P}^m$is the probability of surviving from age *x* to age *x* + *k*, ${q}_{x+k,P}^m$is the probability that an individual aged *x* + *k* will die within the year, ${\mathrm{LE}}_{\mathrm{x}+\mathrm{k}+1,\mathrm{P}}^{\mathrm{m}}$ is LE at age x + k, and ${D}_{x+k,P}^m$is the number of deaths at age *x* + *k*. These elements refer to period P and PI group *m*.

We reject the null hypothesis if the sample value of the statistic ${z}_{x,P}^{m_i-{m}_j}$ is greater than the critical value at a given level of significance *α* in the normal distribution. If that is the case, there is statistically significant evidence that $L{E}_{x,P}^m$ is greater for the higher PI group than for the other.

It is worth indicating that 95% confidence intervals (CIs) of ${\mathrm{LE}}_{\mathrm{x},\mathrm{P}}^{\mathrm{m}}$ are determined by:

8 $$95\% CI\left({LE}_{x,\mathrm{P}}^m\right)={LE}_{x,\mathrm{P}}^m\pm 1.96\bullet \sqrt{VL{E}_{x,P}^m}$$

## Abbreviations

AIME: Average indexed monthly earnings
CI: Confidence intervals
CSWL: Continuous Sample of Working Lives
LE: Life expectancy
PEP: Paid employee workers
PI: Initial pension income
PIA: Primary insurance amount
SE: Socioeconomic
SEP: Self-employed workers
